# Effect of Pin Shape on Thermal History of Aluminum-Steel Friction Stir Welded Joint: Computational Fluid Dynamic Modeling and Validation

**DOI:** 10.3390/ma14247883

**Published:** 2021-12-20

**Authors:** Dmitry Olegovich Bokov, Mohammed Abed Jawad, Wanich Suksatan, Mahmoud E. Abdullah, Aleksandra Świerczyńska, Dariusz Fydrych, Hamed Aghajani Derazkola

**Affiliations:** 1 Pharmaceutical Natural Sciences Department Institute of Pharmacy, Sechenov First Moscow State Medical University, 8 Trubetskaya St., Bldg. 2, 119991 Moscow, Russia; fmmsu@mail.ru; 2 Department of Medical Laboratory Technology, Al-Nisour University College, Baghdad 6770, Iraq; mohammed.a.medical.lab@nuc.edu.iq; 3 Faculty of Nursing, HRH Princess Chulabhorn College of Medical Science, Chulabhorn Royal Academy, Bangkok 10210, Thailand; wanich.suk@pccms.ac.th; 4 Mechanical Department, Faculty of Technology and Education, Beni-Suef University, Beni-Suef 62511, Egypt; iec.mahmoud@gmail.com; 5 Faculty of Mechanical Engineering and Ship Technology, Institute of Manufacturing and Materials Technology, Gdańsk University of Technology, Gabriela Narutowicza Street 11/12, 80-233 Gdańsk, Poland; aleksandra.swierczynska@pg.edu.pl (A.Ś.); dariusz.fydrych@pg.edu.pl (D.F.); 6 Department of Mechanics, Design and Industrial Management, University of Deusto, Avda Universidades 24, 48007 Bilbao, Spain

**Keywords:** friction stir welding, aluminum alloy, St-14 steel, dissimilar joint, computational fluid dynamic, thermal modeling

## Abstract

This article studied the effects of pin angle on heat generation and temperature distribution during friction stir welding (FSW) of AA1100 aluminum alloy and St-14 low carbon steel. A validated computational fluid dynamics (CFD) model was implemented to simulate the FSW process. Scanning electron microscopy (SEM) was employed in order to investigate internal materials’ flow. Simulation results revealed that the mechanical work on the joint line increased with the pin angle and larger stir zone forms. The simulation results show that in the angled pin tool, more than 26% of the total heat is produced by the pin. Meanwhile, in other cases, the total heat produced by the pin was near 15% of the total generated heat. The thermo-mechanical cycle in the steel zone increased, and consequently, mechanical interlock between base metals increased. The simulation output demonstrated that the frictional heat generation with a tool without a pin angle is higher than an angled pin. The calculation result also shows that the maximum heat was generated on the steel side.

## 1. Introduction

The friction stir welding (FSW) process belongs to the group of solid-state joining processes—it enables to provide the appropriate amount of welding activation energy in the form of heat without exceeding the melting point of base materials (unlike fusion processes: arc, plasma, laser and electron beam welding) [1]. This is advantageous because it limits structural transformations and joint properties changes resulting from the crystallization process [2,3]. Compared to traditional joining processes, FSW offers the possibility of making high-strength and low-defect joints, is greener, in many cases cheaper, energy saving and more flexible because there is no need to use consumables and the process can be easily automated [4]. The main limitation of the process is the necessity to select and manufacture a tool of an appropriate shape and made of a material with a higher hardness than the base material. In addition, the elements to be joined must be appropriately fastened [5]. Due to these features, the development of FSW follows the directions of research of various variants of the process limiting individual constraints and developing each of the necessary process conditions: welding machine, tool, the workpiece, process flow, etc. [6,7,8]. Extremely attractive from the point of view of FSW industrial applications is the possibility of using the characteristic properties of various engineering materials by making dissimilar joints, especially from materials that are difficult to weld by conventional fusion welding processes [9,10,11]. For this purpose, it is necessary to overcome technological difficulties caused by significant differences between the materials to be joined in terms of structure, atomic bonds and physico-chemical properties (e.g., melting points, mechanical properties, coefficients of conductivity and thermal expansion) [12,13,14]. A particularly serious problem when joining metals is the formation of intermetallic compounds [15,16] that reduce the plastic properties of joints. Large differences in the properties of aluminum and ferrous alloys make the weldability window extremely limited [17,18]. Despite the simplicity of this process, understanding thermo-mechanical phenomena during welding is complicated. The joint formation mechanisms are based on the formation of a “third body region”: an area heated to the range between recrystallization temperature and melting point of material and characterized by relatively high viscosity and low flow stress [19,20]. Trial and error tests or intelligence systems to optimize final product properties are time-consuming and costly. To overcome these problems, simulation analysis has frequently been used since the 2000s for the FSW process [21,22]. Limited research has reported simulations of dissimilar FSW joints. Among various simulation methods, the computational fluid dynamic (CFD) technique shows significant potential for high accuracy results in the simulation of FSW dissimilar joints [23,24,25]. Besides reported results by various researchers, limited authors considered CFD modeling of the dissimilar joint [26,27]. Difficulty and complexity of equation and boundary conditions, especially at base metal interfaces, caused the simulation of dissimilar joints to be an exciting topic for industries and researchers [28,29,30].

Sundqvist et al. [31] used the CFD model to simulate the thermal history of dissimilar joints between Ti–6Al–4V and AISI 304 L stainless steel. In this research, they used a tool with a frustum pin. Yang et al. used the CFD approach to thermo-mechanical modeling of aluminum and magnesium dissimilar FSW joint. In this research, they used a tool with a cylindrical pin shape. Gotawala and Shrivastava modeled the FSW joint between AA1050 aluminum alloy and copper [32]. They implemented the CFD method for simulation and used a cylindrical pin tool. FSW joining between AA6061 aluminum alloy and Al-Mg2Si composite was simulated with CFD by Sharghi and Farzad [33]. They used a simple cylindrical pin tool to simulate this process. Aghajani Derazkola et al. used a frustum pin to simulate underwater FSW of Al-Mg alloy and low carbon steel [34,35,36,37]. In the available literature on the subject, the results comparing the shapes of the FSW pins during a dissimilar joining have not yet been reported. Using FSW to create new joints between new materials could help many industries improve their structures. In this regard, this paper aims to study the effects of FSW tool pin angle during FSW of AA1100 aluminum alloy and St-14 steel.

## 2. Experimental Procedure

### Raw Materials

In this study, St-14 steel (1.0338) and AA1100 aluminum alloy were selected as base metals. The base metal was provided by a local market and their properties were evaluated and measured in our laboratory. The base materials sheets were cut into small pieces of dimensions 100 mm × 100 mm × 4 mm. The selected materials’ properties are listed in Table 1. The small pieces of base metals were placed in a flexible fixture in order to keep raw sheets fix during welding procedure.

**Figure 1:**
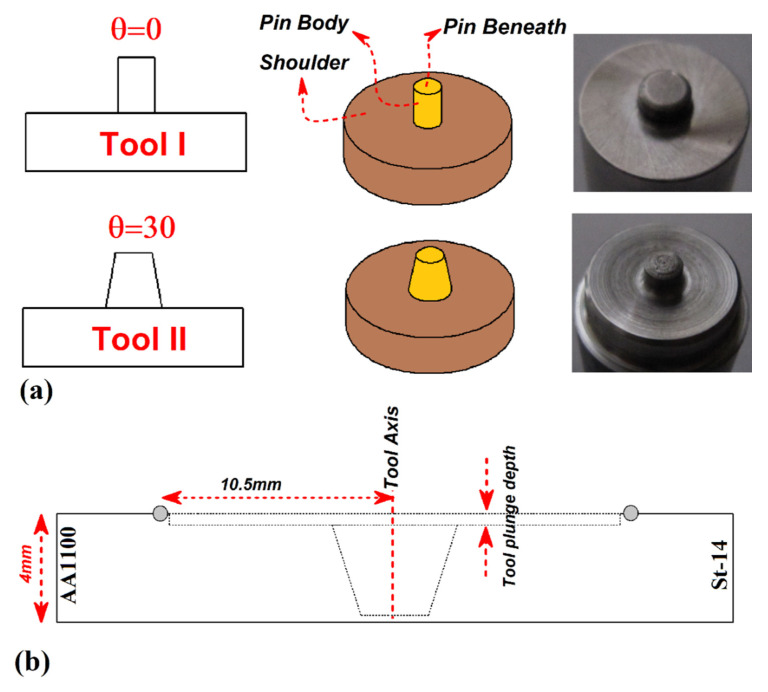
(**a**) Schematic view and image of tool used in this study, (**b**) schematic view of thermocouples placement.

During the experimental procedure, the welding setup was placed in the air so cooling of FSWed samples was at ambient temperature (25 °C). The two different FSW tools were used in this study. The first one had simple cylindrical pin (CP) and second one had frustum pin with 30° trapped angle. Both tools made of tungsten and dimension of used tools are depicted in Figure 1a. During experimental tests, the tool rotational and traverse velocities were 980 rpm and 50 mm/min. For recording of thermal properties during FSW process, two K-type thermocouples (Omega, IL, USA) were placed at near joint line. One thermocouple placed on aluminum side and other placed in steel side. A schematic view from location of thermocouples is depicted in Figure 1b. For investigation of internal materials flow, the FSWed sample was cut from middle and investigated by scanning electron microscopy (SEM) made by Vega-3 Tescan (Prague, Czech).

## 3. Process Modeling

### 3.1. Model Description

A three-dimensional (3D) coupled material flow and heat model was utilized in steady-state conditions in the present study. The simulation procedure was done on the commercially ANSYS FLUENT software under the computational fluid dynamics (CFD) approach. During the simulation process dimensions and geometry of workpieces and tools were according to the actual experimental tests. In the simulation process, the origin point was set at the middle point of the FSW tool shoulder. The x-axis indicated the welding direction, and the z-axis indicated the FSW tool normal axis. The FSW tool had rotational movement, and the interior domain was set to move according to the welding tool speed [38]. The interior domain had the same velocity as the FSW tool traverse velocity. The sidewalls, along with the top and bottom, had the same velocity as the velocity of the inlet. The outer plate of the workpiece was set at zero pressure to avoid the reverse flow at the pressure outlet. The base metals were assumed to be a non-Newtonian single-phase fluid representing the quasi-static thermal and fluid flow boundary problem outside of the interface. In the dissimilar joint case, it was necessary to volume fraction equations at the interface of base metals. In this regard’s two-phase flow conservation equations for continuity, energy and momentum were used to solve the materials mixing at the interface.

### 3.2. Material Model

For modeling the AA1100 aluminum alloy and St-14 steel were selected as the base material (BM). The density and temperature-dependent thermo-mechanical properties were adopted for both BM. As mentioned, both BM were assumed as non-Newtonian fluid, which correlates the deviatoric stress and the strain rate tensors. Non-Newtonian viscosity was assumed to change with the temperature and strain rate [39]. For this reason, the viscosity of each BM (*µ*) as a function of flow stress and strain rate) can be defined [40,41,42]:

(1) $$\mu =\frac{\sigma_{f}}{3\dot{\varepsilon }}$$

The *σ_f_* indicates flow stress of weld metal that can be presented as [43,44,45]:

(2) $$\sigma_{f}=\frac{1}{\alpha }\sinh^{-1}{\left(\frac{Z}{A}\right)}^{\frac{1}{n}}=\frac{1}{\alpha }\left[{\left(\frac{Z}{A}\right)}^{\frac{1}{n}}+\left(1+{\left(\frac{Z}{A}\right)}^{\frac{2}{n}}\right)\right]$$

In Equation (2), *Z* is the Zener–Holloman parameter used for the calculation of the temperature-dependent strain rate [46,47,48,49]:

(3) $$Z=\dot{\varepsilon }\left(\frac{Q}{RT}\right)$$

The *A*, *n*, and *α* are material constitutive constants obtained from the curve fitting of the hot compression test of weld metal at various temperature and strain rates. *Q* and *R* are the activation energy and universal gas constant, respectively. In this regard, the used values for BM used in this study are presented in Table 2:

In the following, the strain rate equation can be calculated by [53,54,55]:

(4) $$\dot{\varepsilon }=\sqrt{\frac{2}{3}\left[{\left(\frac{du}{dx}\right)}^{2}+{\left(\frac{dv}{dy}\right)}^{2}+{\left(\frac{dw}{dz}\right)}^{2}+\frac{1}{2}\left({\left(\frac{du}{dy}+\frac{dv}{dx}\right)}^{2}+{\left(\frac{du}{dz}+\frac{dw}{dx}\right)}^{2}+{\left(\frac{dw}{dy}+\frac{dv}{dz}\right)}^{2}\right)\right]}$$

where *u*, *v* and *w* present the material velocities in the *x*, *y* and *z* directions. With combination Equations (2)–(4), the viscosity of WM can be presented by [1,56,57,58]:

(5) $$\mu =\frac{1}{3\varepsilon \alpha }\left[{\left(\frac{Z\left(T,\alpha \right)}{A}\right)}^{\frac{1}{n}}+\left(1+{\left(\frac{Z}{A}\right)}^{\frac{2}{n}}\right)\right]$$

### 3.3. Boundary Conditions

The heat at interfaces of BM and tool is generated by frictional sliding contact (*Q_f_*) and plastic deformation (*Q_p_*) of both BM during stirring action of tool. In this regard, the heat generation at the interfaces of the FSW tool (shoulder and pin) and each BM can be defined as [59]:

(6) $$Q_{f}=\left[\left(1-\delta \right)\eta \tau +\delta \mu_{f}P_{N}\right]\left(\omega r-U_{1}\sin\theta \right)\frac{A_{r}}{V}$$

where, *A_r_* is any small area on the tool-BM interfaces, *r* is the radial distance of the center of the area from the tool axis, *V* is the control-volume enclosing the area *A_r_*, *τ* is the BM shear stress and *θ* is the angle with the negative x-axis in the counter-clockwise direction, *η* is defined as the mechanical efficiency, *δ* denotes the spatially variable fractional slip between the tool and the BM interface, *µ_f_* is the friction coefficient, *ω* is the rotational velocity, and *P_N_* is the axial pressure. The generated heat by plastic deformation (*Q_p_*), can be calculated as [60]:

(7) $$Q_{p}=\beta \kappa \varphi$$

where *φ* is given by [61]:

(8) $$\varphi =2\overset{3}{\underset{i=1}{\sum }}{\left(\frac{\partial u_{i}}{\partial x_{i}}\right)}^{2}+{\left(\frac{\partial u_{1}}{\partial x_{2}}+\frac{\partial u_{2}}{\partial x_{1}}\right)}^{2}+{\left(\frac{\partial u_{1}}{\partial x_{3}}+\frac{\partial u_{3}}{\partial x_{1}}\right)}^{2}+{\left(\frac{\partial u_{3}}{\partial x_{2}}+\frac{\partial u_{2}}{\partial x_{3}}\right)}^{2}$$

In this equation, *u* indicated materials velocity in *x*, *y*, and *z* direction. In dissimilar joint, *β* is an arbitrary constant that indicates the extent of BM mixing. The *κ* shows the internal mixing (diffusion) of one metal into other metal defined as [50,51,52,62]:

(9) $$\kappa =-V\frac{\partial C_{i}}{\partial x_{1}}+\frac{\partial }{\partial x_{1}}\left(D\frac{\partial C_{i}}{\partial x_{j}}\right)$$

The *V* is plastic material velocity, *C* shows purity of BM at interface during diffusion, and *D* refers to the temperature-dependent chemical diffusion which is defined as [50,51,52,62]:

(10) $$D=A_{1}e^{\left(-\frac{Q_{1}}{RT}\right)}+A_{2}e^{\left(-\frac{Q_{2}}{RT}\right)}$$

**Figure 2:**
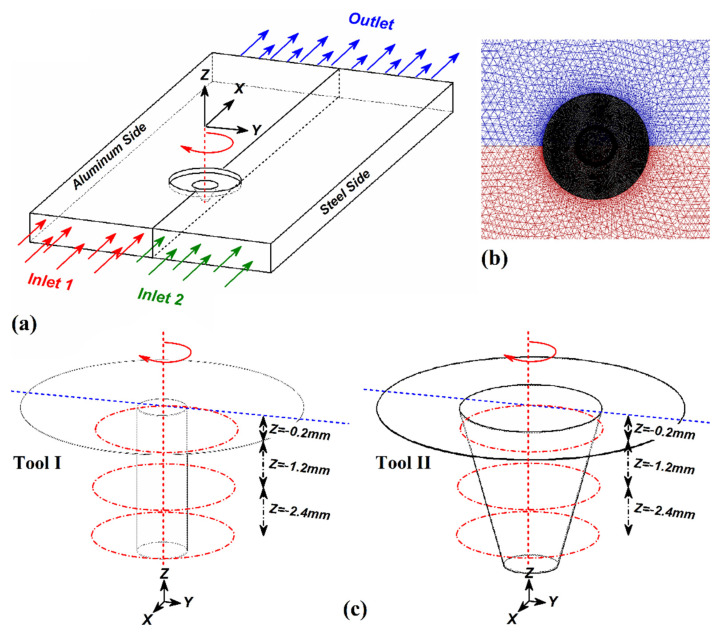
(**a**) FSW simulation domain, (**b**) meshed area, (**c**) schematic view of selected plan for data analysis and their distance from tool shoulder.

With regard to the atomic percent of aluminum and steel at the interface, the parameters chosen for this simulation were defined and showed in Table 3 [50,51,52]:

The FSW domain and meshed process are depicted in Figure 2a,b, respectively. In order to gain a better understanding of simulation results, the FSW tools domain was divided to three areas in height direction (Z). The first area had Z = −0.2 mm distance from tool shoulder, second area had Z = −1.2 mm distance from tool shoulder and finally the last one had Z = −2.4 mm distance from tool shoulder. These areas were used to collect statically results from the simulation. The schematic view of the selected areas is depicted in Figure 2c.

## 4. Results and Discussions

### 4.1. Heat Generation Rate

**Figure 3:**
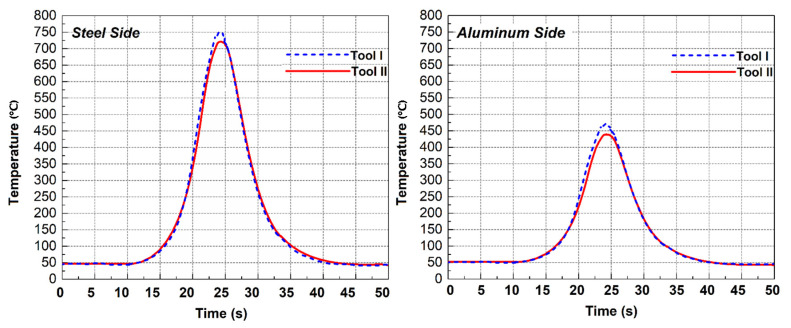
Temperature recorded by thermocouple 1 and 2 at different tool pins.

To better understand the effects of the FSW tool pin angle during dissimilar joining, the heat generation at the different parts of tools are presented in Table 4. The temperature recorded by thermocouples at aluminum and steel sides for both tools are depicted in Figure 3. The obtained results from the simulation show that the tool shoulder generated more heat on the aluminum side compared to steel side for all cases. On the other hand, the tool shoulder generated more heat than the pin on both sides and for both tools. The results revealed that the generated heat in Tool I was higher than Tool II. The results revealed that the lowest temperature was produced at the bottom of the pin. The generated heat by pin body increased from Tool I to Tool II, but in the shoulder area it decreased and caused the total heat generation decreased in Tool II compared to Tool I. The geometrical investigation revealed that the increasing tool pin angle decreases the total joint surface between the Tool and the base metals.

For this reason, the total generated heat in the aluminum and steel sides with Tool II was lower than for the Tool I, which caused the total generated heat by Tool II to be lower. The comparison between experimental results and simulation data showed that the maximum 4% differences were recorded. The maximum recorded by thermocouples was on the steel side for both tools. The maximum recorded temperature for Tool I and Tool II were 772 °C and 761 °C, respectively, while the maximum temperature during the simulation procedure was 738 °C and 725 °C, respectively.

### 4.2. Internal Heat Flow

**Figure 4:**
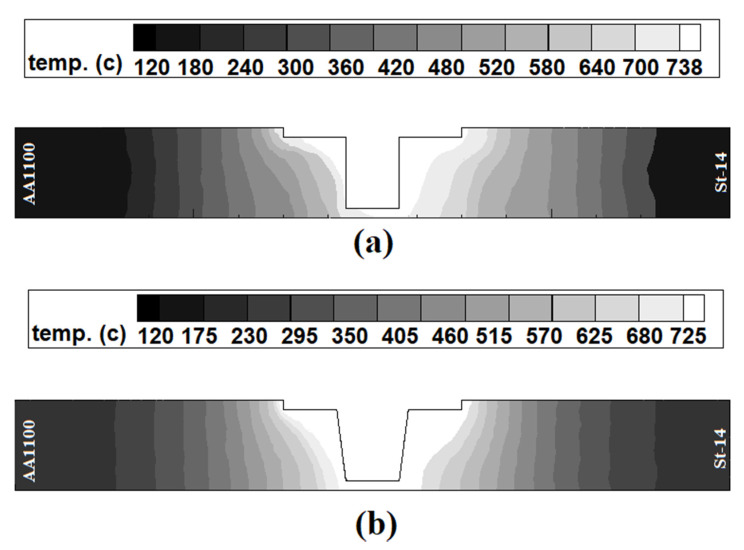
Results of simulation of internal heat distribution in joints made using (**a**) Tool I and (**b**) Tool II.

Figure 4 shows the simulation results of heat distribution from a cross-section view of joints. The results show that the maximum generated heat in joints made using Tool I and Tool II was 738 °C and 725 °C, respectively. These numbers were simulated in the steel side. The heat distribution on the aluminum side and steel side was not equal. Due to the obtained results, the heat concentration on the steel side was higher than on the aluminum side, and it was a common phenomenon. There are two reasons for this issue. First, the generated heat in the St-14 steel side was more than the AA1100 aluminum side. Second, the heat transfer coefficient of AA1100 was higher than the St-14 steel. For this reason, the heat flux in the AA1100 aluminum side was higher than the St-14. This heat flow trend was seen in both joints. On the other hand, the simulation results revealed that the stir zone (hot area) area in the joint made using Tool II was bigger than Tool I. Despite lower generation heat by Tool I, it seems that the stirring action of Tool II was higher than Tool I. The uniform heat distribution shows the uniform mixing of raw metals. The equality of the hot metals’ stirring action leads to symmetry mixing between aluminum alloy and steel. More heat generation and stirring action by Tool II is caused a bigger stir zone and more mixing formed in the joint That FSWed by Tool II.

**Figure 5:**
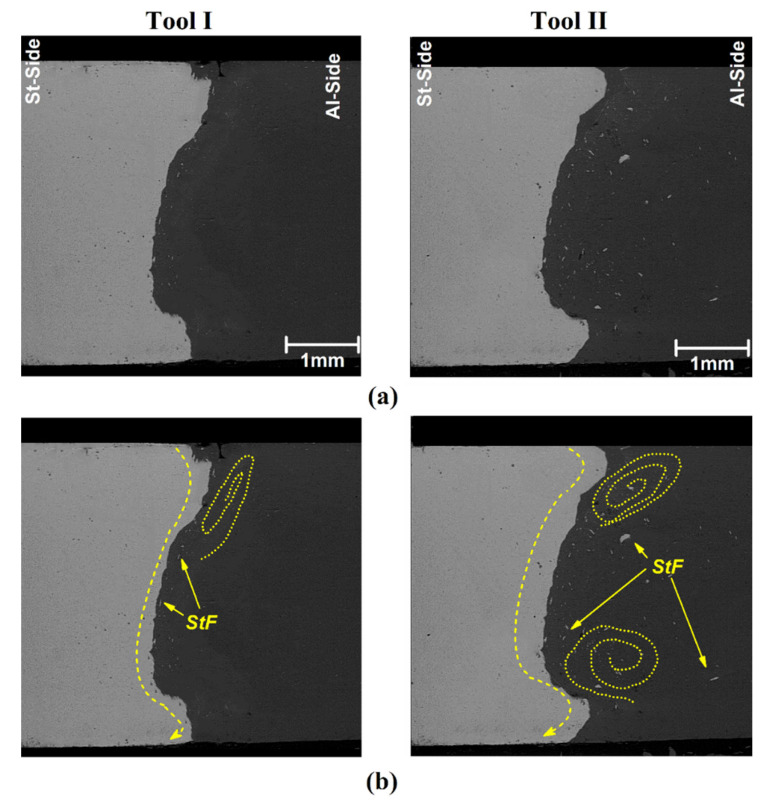
(**a**) SEM images of FSWed sample with Tool I and II, (**b**) internal materials flow pattern that was FSWed with the use of Tool I and II.

To better understand the thermo-mechanical effects of tool pins, the welded samples cut from the middle of the joint line and internal materials’ flow were investigated. The SEM images from the cross-section materials flow of joints welded by Tool I and Tool II are depicted in Figure 5a. The St-14 is seen in grey, and the AA1100 aluminum alloy can be seen in black. The interface of the base metals is not straight and forms a curvy shape. The results indicate that the St-14 steel stretched into the aluminum side and AA1100 alloy and diffused into the steel side from the middle of the joint—this stir zone shape formed in both samples. The wavy shape interface can be seen in the SEM image that shows the mechanical interlocking formed in the interface of base metals [1,55,63,64,65,66,67]. The small hooks formed in the upper and lower area of the stir zone. These hooks were made of St-14 steel. These hooks are seen in both samples. On the other hand, small pieces (fragments) of St-14 can be seen on the aluminum side. It seems that while stirring action of the FSW tool, the steel interface fractured, and small fragments of St-14 spread in the AA100 aluminum side [56,68].

Because the shear strength of AA1100 aluminum is lower than St-14, the FSW tool exerts more stirring action on the aluminum side. It means that the stirring action on the aluminum side was more than the steel side for both cases. On the other hand, the hard steel fragments were inserted into the stirring aluminum and spread in the aluminum matrix. The fractured steel fragments (StF) from the arbitrary edge of steel spread on the aluminum side during the action of base metals with the FSW tool. The produced temperature was far from the melting point of St-14 steel, and for this reason, the StF remain in the AA1100 matrix without any shape-changing or chemical interaction on the macro scale. The SEM image revealed that no lamellar structure or internal voids formed in SZ. The visual inspection from obtained results indicated that the bigger and more StF spread in AA1100, at the joint welded by Tool II.

Due to obtained results, the stirring flow in the joint welded with Tool II is more intense than in the joint that was welded by Tool I (Figure 5b). The swirl flow pattern is detectable in the upper area of the Tool I joint, but this materials flow type is also formed in the up and down area of the joint welded by Tool II. On the other hand, more steel fragments (StF) spread in an aluminum matrix of joints welded by Tool II. The bigger SZ in the joint that was FSWed by Tool II shows that the aluminum matrix’s diffused length of steel fragments was also more. Due to the results, it can be concluded that the mechanical working in the stir zone of the joint that Tool II welded was more intense than for Tool I. For this reason, the bigger stir zone (heated area) was formed in simulation results for Tool II.

### 4.3. Surface Heat Flow

**Figure 6:**
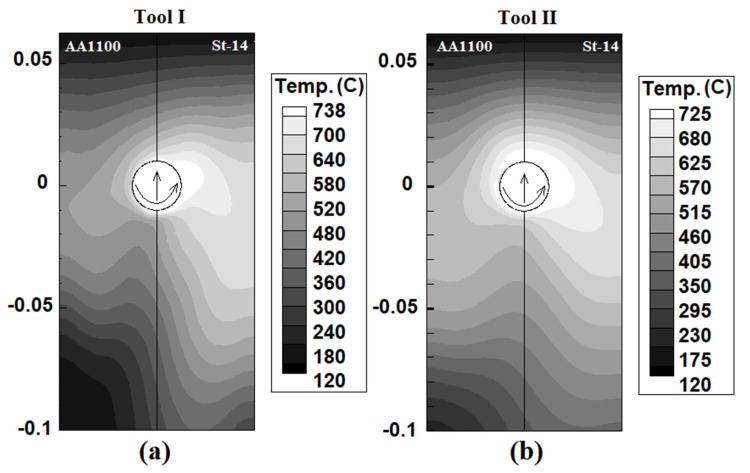
Surface heat flow for joint made with the use of (**a**) Tool I, (**b**) Tool II.

The simulation results from heat distribution on the surface of AA1100 aluminum alloy and St-14 steel during FSW with Tool I and II are depicted in Figure 6. The simulation results are of the surface heat flux following the internal heat distributions. As seen in both cases, the heat concentration on the St-14 side is higher than on the AA1100 aluminum alloy side. As explained, more heat generation on the steel side and lower heat transfer coefficient caused the heat concentration on the St-14 side to be higher than on the aluminum side [69,70,71]. On the other hand, lower heat generation and higher heat transfer coefficient caused the surface heat flux in AA1100 aluminum alloy to be lower than the St-14 steel side. It means the cooling rate of the joint line from the AA1100 aluminum alloy side is higher than from the St-14 steel side.

The higher heat generation obtained by Tool I increased the temperature of base metal surfaces compared to the joint line that was FSWed with Tool II. The comparison between Tool I and II’s surface heat flow revealed that the hot area around Tool II is bigger than around Tool I. In addition, the surface heat distribution in the joint that was FSWed with Tool II is more uniform compared to Tool I. It seems that the higher mechanical action of Tool II made this situation. The stirring action of plasticized metals (as hot masses) in the joint line that was welded by Tool II was higher than Tool I, and for this reason, the desirable area around Tool II is bigger than in the case of Tool I. It indicated that mixing St-14 steel and AA100 aluminum alloy in joint with Tool II was more intense than for Tool I.

### 4.4. Strain Rate and Material Velocity

**Figure 7:**
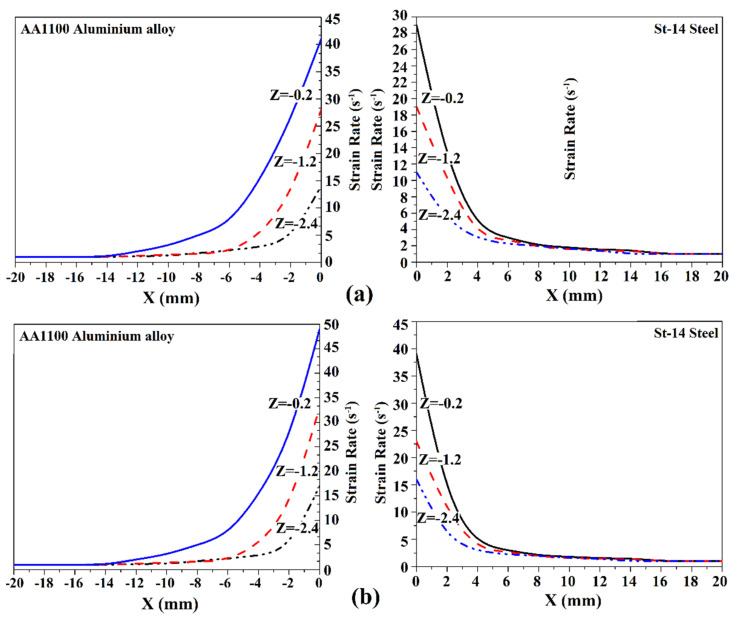
Strain rate at different areas of joint that was FSWed by (**a**) Tool I and (**b**) Tool II.

The static analysis of computed strain rate for Tool I and Tool II pins are presented in Figure 7a,b, respectively. The results were collected from different plans of the pin presented in Figure 2c. The overall survey indicated that the strain rate on the aluminum side was higher than on the steel side in both samples. It seems that the lower strength of AA1100 caused this part to became softer during the FSW process, and stirring action on softer material increased the strain rate. With increasing distance from the shoulder, the strain rate in both materials decreased. This trend shows that at lower areas of the stir zone, stirring action decreases [72,73,74].

On the other hand, the strain rate in the stir zone of the joint that was FSWed by angled pin was more than for Tool I. It seems that the higher application for mechanical works by Tool II increased strain rate in joint line. The maximum strain rate at St-14 steel side simulated 29 S^−1^ and at AA1100 side 42 S^−1^ in the joint by Tool I. The maximum strain rate at St-14 steel side simulated 32 S^−1^ and at AA1100 side 51 S^−1^ in the joint by Tool II. These values were achieved at the top surface of base metals. By moving away from the top surface of workpieces, the heat and mechanical works decreased, and consequently, the strain rate decreased as well.

**Figure 8:**
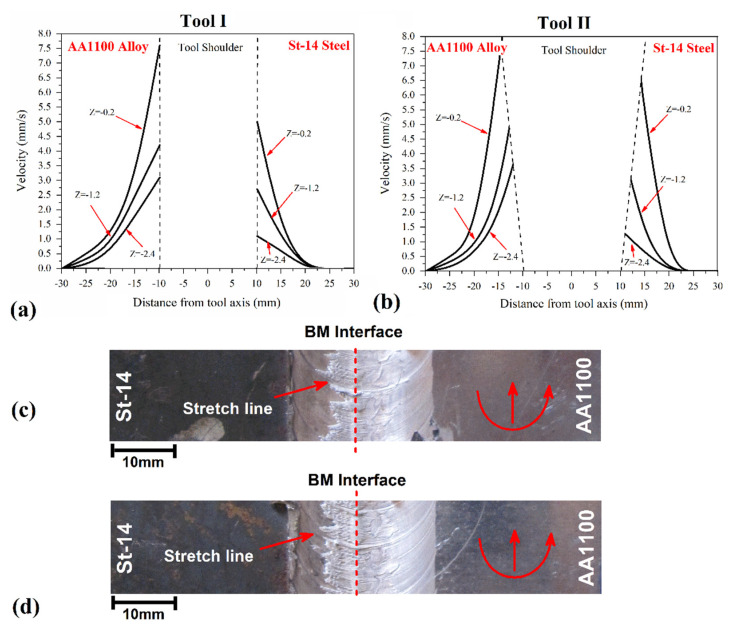
Materials velocity at different areas of joint that FSWed by (**a**) Tool I and (**b**) Tool II. The surface flow of joint that FSWed by (**c**) Tool I and (**d**) Tool II.

In CFD simulation of FSW, materials velocity is relative and related to the tool’s generation of heat and stirring action. The results from the materials’ velocity around both investigated tools are presented in Figure 8a,b. Similarly, with strain rate results, the material velocity results are collected from various plans presented in Figure 2c. The materials velocity in the stir zone is a physical feature that reveals material forging and extrusion around the FSW tool. As the strain rate results revealed, the mechanical action of Tool II was more intense than for Tool I.

For this reason, the strain rate of base materials increased 10% on the aluminum side and 21% on the steel side. In this regard, the velocity of the materials was recorded, and results revealed that the velocity of the material for Tool II was higher than for Tool I. The materials velocity in AA1100 and St-14 steel was not symmetric due to the difference in the base metals’ physical properties, and this phenomenon is seen in both investigated joints. The results show that the maximum material velocity in joints made using Tool I on AA1100 and St-14 steel sides were predicted at 7.4 mm/s and 5.1 mm/s, respectively. The material velocity in joints made using Tool II at AA1100 and St-14 steel sides were predicted 6.4 mm/s and 8.1 mm/s, respectively.

The materials velocity around the tool shoulder determines surface materials mixing [33,75,76]. The surface flow of joints welded by Tool I and Tool II are depicted in Figure 8c,d, respectively. The joint lines formed uniformly, and surface flow rings can be detected in both cases. The main point on the surface of the joint line is the stretching of aluminum alloy into the steel side. The results show that the higher strain rate and materials’ velocity in the Tool II case, which caused more aluminum alloy to stretch into the steel side. This mixing pattern helps to improve the properties of the final weld.

## 5. Conclusions

In this study, the thermo-mechanical aspect of FSW of AA1100 aluminum alloy and St-14 steel was simulated. The effects of FSW tool pin angle on the materials flow and heat generation were investigated by simulation and obtained results are listed as follows:

1- The simulation results of the heat generation of tools indicated that, due to the bigger contact area of tool with cylindrical pin compared to frustum pin with the workpieces, the heat generated by the cylindrical pin tool is higher than for the frustum pin.
2- The internal and surface heat distribution was more uniform in the FSW with the frustum pin tool, and the bigger stir zone formed in the joint with the frustum pin tool due to more stirring action. On the other hand, the generated heat flux on the AA100 aluminum alloy side was more than on the St-14 side.
3- The investigations of the internal materials flow revealed that the tool with frustum pin applied more stirring action in the joint line and caused more mechanical interlock in the interfaces. On the other hand, more steel fragments spread in AA100 aluminum alloy at the joint that was FSWed by frustum pin tool.
4- The maximum strain rate and material velocities were recorded on the top surface of workpieces. The maximum value of the strain rate in the joint that was FSWed with the frustum pin was 21% (aluminum side) and 10% (steel side) more than for the joint that was FSWed with the cylindrical pin.

## Figures and Tables

**Table 1:** Mechanical properties of base metal.

Parameter	AA1100 Aluminum Alloy	St-14 Steel
*ρ* (kg/m^3^)	2710	7810
Melt point (°C)	657	1400
σ_Y_ (MPa)	34	344
σ_UTS_	90	580
τ (MPa)	62	360
Elongation (%)	35	15

**Table 2:** Values of parameters of base metals.

Parameter	AA1100 [50,51,52]	St-14 [50]
*Q* (kJ/mol)	158.3	204
*R* (J/K·mol)	8.314	8.314
*A* (1/S)	5.18 × 10^10^	0.62 × 10^10^
*n*	5.66	1.18

**Table 3:** Chemical parameters of workpieces.

Parameters [50,51,52]	Value [50,51,52]
*Q*_1_ (kJ/mol)	280.5
*Q*_2_ (kJ/mol)	276.3
*A*_1_ (cm^2^/s)	148.1
*A*_2_ (cm^2^/s)	60.3
*C*_1_ (Fe purity at interface (at. %))	50
*C*_2_ (Al purity at interface (at. %))	50

**Table 4:** Area of various part of tools and related generated heat.

	Area (m^2^)		Generated Heat on Aluminum Side (°C)		Generated Heat on Steel Side (°C)	
	I	II	I	II	I	II
Shoulder	0.02859	0.02721	331	304	517	475
Pin Body	0.00942	0.01055	68	77	132	161
Pin Beneath	0.00283	0.00283	62	62	89	89
Total	0.04084	0.04059	461	443	738	725

## Data Availability

Data sharing is not applicable to this article.
